# Process evaluation protocol of a trial evaluating app-based post knee arthroplasty telerehabilitation strategy in India

**DOI:** 10.12688/wellcomeopenres.26007.1

**Published:** 2026-04-08

**Authors:** Siaa Girotra, Purnima Shrivastava, Sandhya Kanaka Yatirajula, Devarsetty Praveen, Ruchika Madan, Seema Grover, Ajit Kumar, Sahil Batra, Bhavuk Garg, Rajesh Malhotra, Ralph Maddison, Niveditha Devasenapathy

**Affiliations:** 1 The George Institute for Global Health India, New Delhi, Delhi, India; 2 The George Institute for Global Health, Camperdown, New South Wales, Australia; 3 Prasanna School of Public Health, Manipal, Karnataka, India; 4 Department of Geriatric Medicine, All India Institute of Medical Sciences, New Delhi, Delhi, India; 5 Department of Physiotherapy and Rehabilitation, Indraprastha Apollo Hospitals, New Delhi, Delhi, India; 6 Department of Orthopedics, All India Institute of Medical Sciences, New Delhi, Delhi, India; 7 Department of Orthopedics, Indraprastha Apollo Hospitals, New Delhi, Delhi, India; 8 Deakin University Institute for Physical Activity and Nutrition, Geelong, Victoria, Australia

**Keywords:** Arthroplasty, Knee Replacement, Mobile applications, Telemedicine, Rehabilitation, Process Assessment

## Abstract

**Background:**

Physical rehabilitation after knee arthroplasty is essential for improving functional outcomes. However, adherence to rehabilitation is generally poor. We developed a multicomponent mobile application (TeleREhabilitation after knee ArThroplasty; TReAT app) to facilitate home-based rehabilitation. The TReAT trial is a 1:1 randomized controlled superiority trial to evaluate the clinical and cost-effectiveness of the intervention against usual care, with an embedded process evaluation.

**Methods and analysis:**

Consenting individuals undergoing knee arthroplasty at a public and private tertiary care hospital in Delhi with the ability to use a smartphone were randomized to app-based or usual home-based rehabilitation and followed up at 3 and 6 months. TReAT is an mHealth intervention consisting of provision of education and exercise videos, an e-diary for self-monitoring of progress, two-way communication, and remote therapy planning by healthcare providers. Usual care varies by hospital and consists of oral or written instructions regarding rehabilitation. We use a mixed-methods convergent parallel design for process evaluation and will draw upon backend app analytics, interviews with end users, and researcher notes and observations as data sources. We will present descriptive summary for reach and effectiveness outcomes and emerging themes of qualitative data for acceptability, maintenance and context. Mixing will occur at the data collection and interpretation stage for fidelity, usage by end users, adoption, and mechanistic pathways of the various components of the intervention.

**Conclusions:**

The process evaluation will provide insights into the probable uptake and scalability of TReAT intervention. The contrast of findings between public and private health care settings will inform further understanding of how the intervention plays out in different settings. The findings will guide the development of implementation strategies to improve rehabilitation care using mHealth interventions in India.

**Trial registration:**

The trial was registered prospectively with the Clinical Trial Registry of India (CTRI/2024/06/068838).

## Plain Language summary

This study outlines the methodology for assessing how well a mobile application-based intervention for post-knee replacement care is implemented in a research setting. Furthermore, this paper describes how the acceptability of the intervention will be captured along with contextual factors that may impact uptake by end users. This evaluation of the intervention implementation is performed alongside an experimental study that compared mobile application-based intervention with usual home-based rehabilitation care. This study, conducted at two tertiary care hospitals in Delhi, India, will utilize observations from researchers and interviews with end users who are patients, family members, and healthcare providers. The results from this evaluation will help to interpret the results of the trial and gauge the scalability of the intervention in a real-life setting. This methodology paper might be of interest to researchers working in rehabilitation mHealth and implementation sciences.

## Introduction

Physical rehabilitation after knee arthroplasty is essential to achieve optimal functional outcomes. ^1^ Poor adherence to rehabilitation protocols can lead to suboptimal outcomes or delayed recovery. ^2^ Behaviour change interventions hold the potential to improve adherence to physical rehabilitation. ^3, 4^ Bandura’s self-efficacy theory postulates that a person’s belief in their ability to perform a task serves as a motivation for performing the task. ^5^ Interventions aimed at improving this belief in self-ability can help improve the recovery trajectory of individuals after knee arthroplasty. ^6^

In line with Bandura’s theory of self-efficacy, we designed and developed a mobile-based health (mHealth) intervention using the behavior design thinking approach to support home-based rehabilitation. ^7^ The overarching aim of this intervention is to detect slow recovery, improve adherence to prescribed rehabilitation regimens, and reduce the need for clinical visits. The intervention consists of five interlinked components that provide information to patients about the recovery pathway following knee arthroplasty, enable the patient to adhere to the prescribed exercises via reminders, motivational messages, and recorded exercise videos; a secure portal for one-to-one communication with health care providers via text, video, and audio calls; and self-management through use of an e-diary. Furthermore, this intervention enables healthcare providers to remotely monitor and prescribe personalized physical therapy plans. ^7, 8^ This intervention is delivered through the “TeleRehabilitation after knee ArThroplasty (TReAT) app” which consists of seven functionalities, exercise videos, e-diary, knowledge material, progress graphs, in-app messaging, in-app video consultation and a tool to self-measure knee range of motion. The details of the app functionalities are illustrated in **section 1 of the supplementary file.** ^9^

The clinical and cost-effectiveness of this app-based rehabilitation support is being evaluated in an open-label, 1:1 randomized, superiority trial (TReAT trial) at two tertiary care hospitals. ^10^ Consenting individuals with the ability to use the basic functions of a smartphone and willing to visit at 3- and 6-months post knee arthroplasty are randomised to either the TReAT app plus usual care or usual care only. The intervention is provided for a minimum duration of 6 months. In India, usual care after knee arthroplasty consists of unsupervised home-based rehabilitation, which consists of provision of oral or written instructions at discharge and follow-up check-ups with the surgeon or physiotherapist as per individual need. Individuals may avail support from physiotherapy services at home or visit a clinic at their discretion. To understand the implementation fidelity, reach, any unanticipated additional activities or adaptations to the intervention, and the mechanistic pathway of various components of the TReAT intervention, we are conducting a process evaluation using a mixed-methods approach in accordance with the UK Medical Research Council process evaluation guidance for complex interventions. ^11^ In this study, we detail the process evaluation protocol for the TReAT trial.

### Objectives

• To evaluate implementation *fidelity* (how and extent of implementation with the original plan)
• To assess the *reach* (participants’ enrolment and retention) of the intervention, *dose* of the intervention *delivered and received*, and *usage* of the application
• To identify *contextual factors (barriers and facilitators)* for implementing this intervention
• To examine user *satisfaction and acceptability* of intervention components
• To examine the correlation between the extent of app function usage and expected behavioral changes *(mechanistic pathway)*

## Methods

### Conceptual framework

The process evaluation plan was developed in accordance with the Linnan and Steckler theoretical framework ^12^ adapted from Saunders et al. ^13^ and tailored to TReAT intervention. The process evaluation outcomes are based on the RE-AIM framework ^14^ and the implementation outcome framework. ^15^

**Figure 1.**
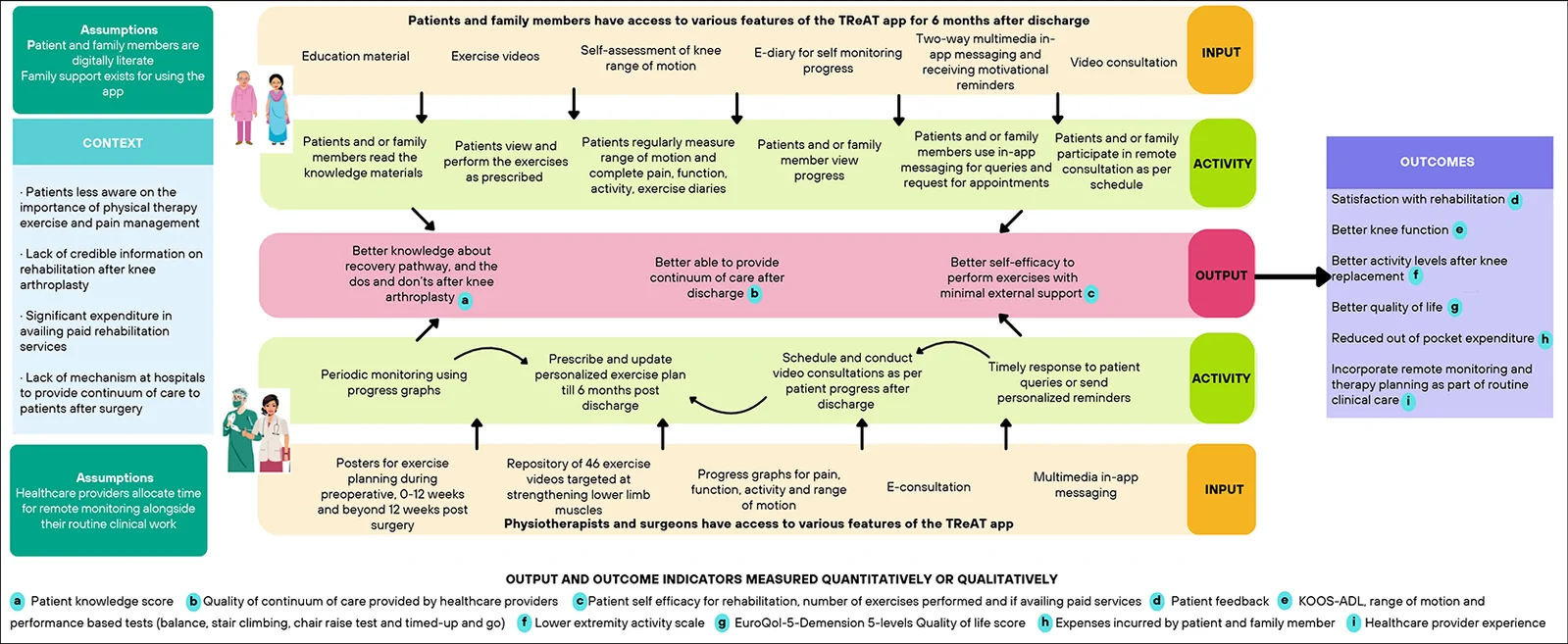
Intervention logic model. Legend- The figure illustrates the hypothesized mechanism of change enabled by the app-supported rehabilitation. The ‘Context’ is based on findings from literature review and formative phase of this project.

A thematic illustration of the mechanism of change, using a logic model, is shown in Figure 1. We hypothesized that providing access to educational material from a credible source would improve patient knowledge about the recovery process and assist in goal setting for the return to routine activities of daily living. System-generated notifications, self-monitoring progress, and periodically communicating with healthcare providers through app messaging and video consultation would improve their self-efficacy to adhere to the rehabilitation protocol. Personalized exercise therapy prescribed based on progress gauged either by remote consultation or by patient diary entry can help achieve better pain management and knee function. Thus, regular communication between healthcare providers and patients would aid in providing a continuum of care and identifying any modifiable reasons for slow recovery.

We defined ideal implementation of the intervention to include the following: (i) successful installation of the application by the end users with or without assistance from the research coordinator, followed by training on the use of different app components; (ii) healthcare providers use specific components of the app; for example, a physiotherapist and surgeon will monitor patient progress, update the therapy plan (minimum of 3 updates after discharge), and schedule video consultations (minimum 1 video consultation/per month in 0-3 months) as per patient requirements; (iii) patients and family members would be reading the educational material, referring to exercise videos, using the e-diary for self-monitoring (at least 50% as advised) and communicating with the healthcare providers via messaging and/or video consultation would indicate that the intervention is acceptable.

### Study setting

The trial is being conducted at a public and private tertiary care hospital in Delhi, India, which will provide a contextual contrast in implementing the intervention. Individuals scheduled for or undergoing knee arthroplasty will be eligible for the trial if they own a smartphone and are able to use their basic functionalities. Furthermore, they should be willing to visit the hospital at 3 and 6 months after enrolment. The first participant was enrolled in June 2024 and recruitment ended in December 2025. The overall planned trial sample size was 300 participants.

### Study design

**Figure 2.**
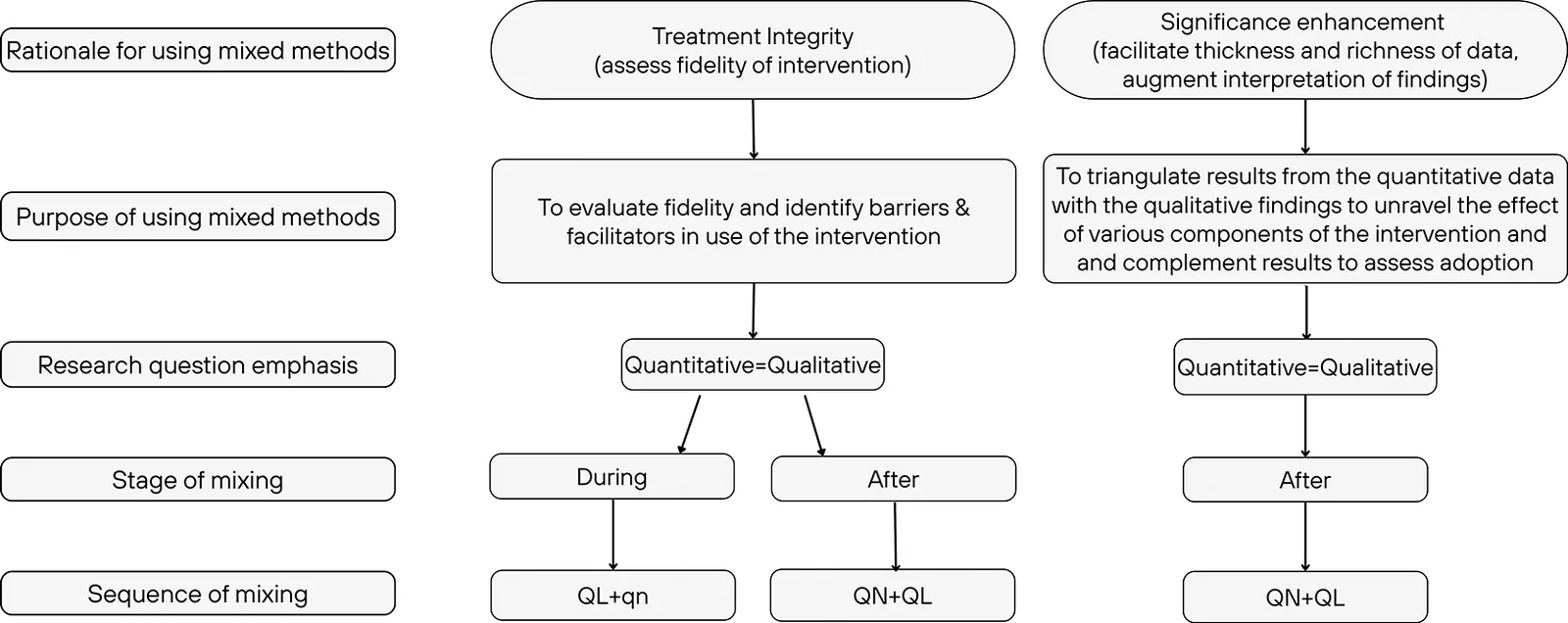
Rationale and purpose of mixed methods approach. Legend- Note: QN= quantitative, QL=qualitative, ‘+’= concurrent, ‘uppercase’= dominant, ‘lowercase’= less dominant. Reference: Collins KM, Onwuegbuzie AJ, Sutton IL. A model incorporating the rationale and purpose of conducting mixed-methods research in special education and beyond. Learning disabilities: A contemporary journal. 2006;4(1):67-100.

We will use a mixed-methods approach to collect data, analyze and interpret the results ^16^ using a convergent parallel design with nested sampling ( Figure 2). The rationale for using the mixed-method approach is “treatment integrity” and “significance enhancement”. ^16^ Under treatment integrity, the purpose was to identify discrepancies between planned and actual implementation of the intervention and identify barriers and facilitators in using the app. Under significance enhancement, the purpose is “triangulation” to understand the mechanism by which the intervention components lead to the outcome and “complementarity” to assess the adoption of the intervention by comparing results obtained from quantitative and qualitative methods. The participants for the process evaluation were chosen from those participating in the trial and are elaborated in the subsequent sections.

### Outcomes and data sources

We will assess reach, implementation fidelity, effectiveness, extent of use, acceptability and adoption of the intervention in the context in which the trial is conducted as process evaluation outcomes. We will measure the output level indicators to understand the mechanistic pathways by which the intervention components will lead to the desired outcome. Definitions of these outcomes are available **in section 2 of the supplementary file.** ^9^ All randomized participants will contribute to reach and effectiveness; however, only the TReAT app group will contribute to implementation fidelity, extent of use, acceptability, appropriateness, and maintenance. Healthcare providers (surgeons and physiotherapists) will contribute to the understanding of TReAT adoption during trial and routine practice. The source of data for the outcomes will be backend analytics of the mobile app, interviews with patients, physiotherapists, and surgeons, and researcher notes and observations ( Table 1).

**Table 1.** Description, measurement and analytic approach of the outcomes.

Outcome	Key questions (methodology)	What is measured and how is measured?	Analysis
Reach and Penetration	What was the extent to which those who were approached and eligible received the intervention? (Quantitative)	Number approached, ineligible, refused and recruited with their demographic characteristics (age, gender, literacy and location of residence) using a patient screening log	Reach=number recruited in the trial/total approached Penetration=number recruited in the trial/total eligible
Effectiveness	What was the difference in knee function and quality of life between intervention and control group? (Quantitative)	Self reported Knee function score (KOOS-ADL) and quality-of-life (EQ-5D-5L) of trial participants in both groups measured at 3 and 6 months	Group difference in KOOS-ADL and quality of life scores at 3 and 6 months
Pathways to outcome( Which components of the intervention could have impacted the outcome?)	Does provision of education material and exercise videos improve knowledge about post-op care, rehabilitation adherence and number of exercises performed? Does knowledge and adherence vary by extent of app use? Does increase in knowledge lead to better function? (Quantitative and Qualitative)	Extent of app used from the fidelity tool. Patients’ knowledge about recovery (14-item questionnaire), rehabilitation adherence (22-item questionnaire) and exercise performance using a predefined checklist. These are study specific tools administered to all participants at 3 months.	Group difference of patient knowledge score, rehabilitation adherence score and proportion of exercise performed at 3 months. Difference in these scores by use of the app components. Correlation of these scores with KOOS-ADL scores by group.
Fidelity	How was the intervention delivered and to what extent was the intervention delivered as it was planned? (Qualitative and Quantitative)	Intervention components used by end users captured from researcher notes, app analytics data compiled using a fidelity tool	Proportion of patients who used each component. Proportion of patients for whom a specific component was used by healthcare providers.
Adoption	How did the healthcare providers uptake the intervention during the study? (Qualitative and Quantitative)	Healthcare provider engagement with the application for providing post knee arthroplasty rehabilitation care captured via unobtrusive observations, healthcare provider interviews, and app analytics	Proportion of patients for which app components were used in each context and compare it with the organisational and provider level barriers and facilitators identified. Quality of remote interactions *
Usage	What was the experience of the end users (challenges, perceived benefits) in using the TReAT app (Qualitative and Quantitative)	Researcher notes and app analytics data Semi-structured interview of end users (during and end of the intervention period)	Frequency of use of each component of app and thematic analysis for factors related to use and non-use of the app
Acceptability and appropriateness	How satisfied were the end users with the intervention? (Qualitative)	Semi-structured interviews of end users to know satisfaction with navigating the app, satisfaction with the contents of the intervention and its perceived relevance	Thematic analysis for contextual factors at patient and provider level.
Context	Did any individual level characteristics and health sector (public and private) influence implementation at each site? What were the context specific adaptations that were made to address emerging challenges? (Qualitative)	Semi-structured interview of healthcare provider at the end of the study and researcher notes maintained throughout the study	Thematic analysis for contextual factors at organisational, provider and patient level.
Maintenance	What is the healthcare providers’ perception on the potential for routine use of such mHealth interventions in rehabilitation care? (Qualitative)	Semi structured interviews with healthcare providers towards the end of the trial to assess health care providers buy-in and opinions on the potential for use of the intervention in routine care	Thematic analysis for contextual factors at organisational and provider level.

* Quality of healthcare provider’s interaction in video consultations will be assessed by an observer unobtrusively to gauge whether the interaction motivated the patient to follow the rehabilitation instructions, be able to address the health-related queries, remind them to refer the app for information, and examine wound healing, gait, and range of motion remotely.

Backend analytics from the mobile app: Participants’ and healthcare providers’ usage and adherence to the intervention will be assessed using data from the backend app analytics. The data include the number of times the patients complete the diary (pain, exercise, knee function, and activity), the exercise prescription is modified by the healthcare provider, and video consultations scheduled. Use of the app by family members, number of times exercise videos are viewed, information about accessing the educational material, frequency of using the messaging feature, and viewing progress graphs are not available in the app analytics.

Screening log: A screening log maintained by the research coordinator at each study site will document the number of patients scheduled for knee arthroplasty, number approached, enrolled, reasons for ineligibility, and refusal to participate, along with age, sex, literacy, and state of residence.

Researcher notes: The research coordinator will document information related to app installation, healthcare provider, and patient engagement with the app and the quality of video calls in a structured diary using an Excel workbook from direct observations and periodic checks in calls made to the trial participants ( **section 3 & 4 of the supplementary file** ^9^).

End-user interviews will be conducted to explore participant experience with remote consultation, usefulness of knowledge material, satisfaction with the app, and use of the app to support rehabilitation. The interviews will be conducted with a random subsample of participants in the intervention group stratified by app usage. Participants will be classified as full, partial, or non-users based on the number of components of the app used. Participants who use more than five of the seven components of the app will be classified as full users. Partial users will be those who use at least one to five components, and nonusers will be those who failed to install or did not use the app even after installation. Physiotherapists and surgeons participating in the trial will be interviewed at the end of the study to obtain feedback on their experience in app usage and the potential for uptake of this intervention in routine rehabilitation care. Semi-structured interview guides for the interviews are available in **section 5 & 6 of supplementary file.** ^9^

Study specific questionnaires: Patients’ knowledge about recovery, the type and number of exercises being performed, and rehabilitation adherence will be measured at 3 months in both groups to understand the mechanisms through which the intervention may have an impact on the functional outcomes ( **section 7, 8 & 9 of supplementary file** ^9^).

### Ethical considerations

The trial protocol, including the process evaluation activities, was approved by the ethics committees of the sponsor institute and clinical sites. Written informed consent was obtained from all study participants.

### Data collection

Quantitative component: Data related to knowledge, exercise performance, and rehabilitation adherence will be collected by trained research coordinators at the 3-month follow up using study-specific questionnaires. Data will be collected on paper forms and entered into a web-based electronic data capture system (REDCap) with built-in validation checks and a query management system. Researcher notes and observations will be collated in a structured Excel spreadsheet ( **section 10 of supplementary file** ^9^ **)**.

Qualitative component: Interviews will be conducted during the 6-month follow-up from purposively selected participants in the app-based rehabilitation group (users, partial users, non-users based on the app analytics and researchers’ notes) using a semi-structured topic guide to explore the rehabilitation experience and barriers and facilitators to intervention use. Healthcare providers will be interviewed to explore their experience in using the app and their perceptions of the potential for this intervention in routine rehabilitation care. Interviews will be conducted by a researcher trained in qualitative interview methods, either face-to-face or telephonically, and audio recorded. If participants are uncomfortable or refuse to participate in the audio recording, notes will be taken during the interview by the researcher. Participants will be invited to participate in the interviews until data saturation is reached for each category.

### Data management

Data from questionnaires related to process evaluation are entered into the trial database developed in REDCap and will follow the same procedures for ensuring data quality, confidentiality, and security as for the effectiveness outcomes, as described in detail in the trial protocol. ^10^ Data from backend app analytics will be periodically extractable by the trial team without personal identifiers. Qualitative interviews will be transcribed and translated by one member of the research team and cross-checked by another member of the team for correctness and appropriateness. All patient identifiers will be removed at the time of the transcription. Data related to the screening log and researcher notes maintained in an Excel sheet will be stored only for a definite duration after trial completion. The data on the app platform will be destroyed five years after the completion of the trial. The rest of the data will be stored securely with restricted access on the sponsor’s server, as per institutional policy. The paper forms and informed consent will be safely secured in a cabinet with restricted access at the sponsor institute as per the institutional policy.

### Data analysis

Quantitative: The proportion of non-participants and participants recruited from the total approached (reach) and recruited of the total eligible (penetration) groups will be presented along with sociodemographic characteristics. App usage by patients (use of diary, use of messaging feature, video consultation, using videos to perform exercises, measuring knee range of motion using the app) and frequency of use will be presented. App usage by healthcare providers will be presented as the proportion of patients for whom they used the app functions, such as progress graphs, modification of exercise therapy plans, and remote consultations.

Qualitative: Transcripts of qualitative interviews will be coded independently by two researchers in NVivo software (Lumivero (2023) NVivo (Version 14)) using an inductive-deductive approach followed by thematic analysis. The codes will be compared for variation and similar ideas will be grouped into categories before organizing them into overarching themes related to facilitators and barriers in an iterative manner.

Mixed methods: Data will be mixed at both the data collection and interpretation stages. At collection, participants will be grouped into pre-specified categories: users, non-users, and partial users. The data for this categorization will be based on researcher notes and application analytics. At interpretation, researcher notes will be utilized to understand why certain components of the app were used more frequently. Organizational and provider-level barriers will be compared with the proportion of patients monitored remotely to decipher the contextual factors that may affect the adoption of the intervention. The mechanistic pathway will be explored by comparing the group difference of output indicators between the intervention and control groups and examining the correlations between output and outcome indicators by intervention group and within the treatment group based on the extent of use. the analytical approach for each outcome is presented in Table 1.

## Discussion

This paper describes the design of a mixed-method process evaluation protocol for a multicomponent behavioral intervention conducted alongside a randomized controlled trial. ^10^ The TReAT intervention was designed to support recovery and rehabilitation of patients undergoing knee arthroplasty. There is an increasing trend of mHealth interventions being developed and evaluated for patient management in clinical contexts, but details about intervention fidelity, adoption, and implementation ability are often understudied or under-reported, limiting the interpretation of trial findings. ^17, 18^

This evaluation, guided by the Linnar and Strickler framework ^12^ will help us understand the results of the study, irrespective of the strength and direction of our findings. We will also be able to answer questions regarding the generalizability and future scalability of this intervention in populations with less proficiency in using smartphones and low-resource settings. The remote monitoring approach using mobile applications may not be novel in the global context ^19^ however, the contextual factors influencing the adoption of the remote monitoring approach among patients and healthcare providers will add valuable information for future work in this space in a low-middle-income country context.

### Strengths and limitations

The strengths of this evaluation lie in its comprehensive planning and clear reporting of the mixed-methods approach. We will be reporting the results of this evaluation following the standard reporting guidelines of mixed-methods studies for rehabilitation and health sciences. ^20^ The integration of qualitative and quantitative methods will provide a more nuanced understanding of the findings and lead to better use of the data collected. A limitation of the study is that the findings will be more applicable to tertiary care referral hospitals in the North Indian context. Second, reliance on researcher notes and self-reported data may introduce reporting bias.

## Conclusion

This protocol is an attempt to improve the planning, analysis, and interpretation of process evaluation, alongside a clinical trial evaluating an mHealth intervention for remote monitoring. The results of this trial will inform future mHealth interventions in physical rehabilitation spaces in the context of the elderly population and lower-middle-income countries.

## Patient and public involvement

There was no patient or public involvement in the design or conduct stages of the process evaluation of this trial.

## Data Availability

No data are associated with this article The supplementary file and completed SPIRIT 2025 checklist can be found in **Open Science Framework** in a folder named “Process evaluation protocol” in the project ‘Telerehabilitation for post knee replacement monitoring (TReAT project).’ This file can be accessed at https://doi.org/10.17605/OSF.IO/AQGRM ^9^ Description of data: This supplementary file contains a description of the intervention, data collection tools, and definitions of the implementation outcomes.
1. eFigure: TReAT app to deliver the intervention 2. eTable: Definitions of process evaluation outcomes 3. Structured format to capture researcher’s notes for the intervention group 4. Structured format to capture researchers note-usual care group 5. Semi-structured interview of patients or family member-intervention group 6. Semi-structured interview guide for health care providers (surgeons and physiotherapists) 7. Questionnaire to assess patients’ knowledge about recovery 8. Questionnaire to assess rehabilitation adherence 9. Questionnaire to assess exercise performance 10. Fidelity tool Data are available under the terms of the Creative Commons Zero “No rights reserved’ data waiver (CC0 1.0 Public domain dedication).
