# Process evaluation protocol of a trial evaluating app-based post knee arthroplasty telerehabilitation strategy in India

**DOI:** 10.12688/wellcomeopenres.26007.3

**Published:** 2026-09-29

**Authors:** Siaa Girotra, Purnima Shrivastava, Sandhya Kanaka Yatirajula, Devarsetty Praveen, Ruchika Madan, Seema Grover, Ajit Kumar, Sahil Batra, Bhavuk Garg, Rajesh Malhotra, Ralph Maddison, Niveditha Devasenapathy

**Affiliations:** 1 The George Institute for Global Health India, New Delhi, Delhi, India; 2 The George Institute for Global Health, Camperdown, New South Wales, Australia; 3 Prasanna School of Public Health, Manipal, Karnataka, India; 4 Department of Geriatric Medicine, All India Institute of Medical Sciences, New Delhi, Delhi, India; 5 Department of Physiotherapy and Rehabilitation, Indraprastha Apollo Hospitals, New Delhi, Delhi, India; 6 Department of Orthopedics, All India Institute of Medical Sciences, New Delhi, Delhi, India; 7 Department of Orthopedics, Indraprastha Apollo Hospitals, New Delhi, Delhi, India; 8 Deakin University Institute for Physical Activity and Nutrition, Geelong, Victoria, Australia

**Keywords:** Arthroplasty, Knee Replacement, Mobile applications, Telemedicine, Rehabilitation, Process Assessment

## Abstract

**Background:**

Physical rehabilitation after knee arthroplasty is essential for improving functional outcomes. However, adherence to rehabilitation is poor. We developed a multicomponent mobile application TeleREhabilitation after knee ArThroplasty (TReAT app) comprising education, exercise videos, an e-diary for self-monitoring progress, two-way communication, and remote therapy planning to facilitate home-based rehabilitation. The TReAT trial is a 1:1 randomized controlled superiority trial to evaluate the clinical and cost-effectiveness of the intervention against usual care, with an embedded process evaluation. This paper reports the protocol for process evaluation.

**Methods and analysis:**

Consenting individuals undergoing knee arthroplasty at a public and private tertiary care hospital in Delhi, who are able to use a smartphone will be randomized to either app-based or usual home-based rehabilitation and followed up at 3 and 6 months to assess knee-related functional outcomes. A mixed-methods convergent parallel design supported by a logic model will be used for a process evaluation. Data from screening log, backend app analytics, interviews with end users, and researcher notes will contribute to the evaluation based on RE-AIM framework. Researcher notes will be quantified and, consolidated with app analytics to summarise fidelity, and adoption. Thematic analysis of interviews will provide insights into contextual factors influencing intervention adoption during the trial and intention to use the TReAT app beyond the trial period. To explore potential association between use of intervention components and outcomes, correlations between functional outcomes, knowledge score and rehabilitation self-efficacy will be examined.

**Conclusions:**

The process evaluation will provide insights into the potential uptake and scalability of TReAT intervention. Comparing findings across public and private healthcare settings will enhance understanding of how the intervention functions in different contexts. The findings will inform implementation strategies to improve rehabilitation care using mHealth interventions in India.

**Trial registration:**

The trial was registered prospectively with the Clinical Trial Registry of India (CTRI/2024/06/068838).

## Plain Language summary

This study outlines the methodology for assessing how well a mobile application-based intervention for post-knee replacement care is implemented in a research setting. Furthermore, this paper describes how the acceptability of the intervention will be captured along with contextual factors that may impact uptake by end users. This evaluation of the intervention implementation is performed alongside an experimental study that compared mobile application-based intervention with usual home-based rehabilitation care. This study, conducted at two tertiary care hospitals in Delhi, India, will utilize observations from researchers and interviews with end users who are patients, family members, and healthcare providers. The results from this evaluation will help to interpret the results of the trial and gauge the scalability of the intervention in a real-life setting. This methodology paper might be of interest to researchers working in rehabilitation mHealth and implementation sciences.

## Introduction

Physical rehabilitation after knee arthroplasty is essential to achieve optimal functional outcomes. ^1^ Poor adherence to rehabilitation protocols can lead to suboptimal outcomes or delayed recovery. ^2^ Behaviour change interventions hold the potential to improve adherence to physical rehabilitation. ^3, 4^ Bandura’s self-efficacy theory postulates that a person’s belief in their ability to perform a task serves as a motivation for performing the task. ^5^ Interventions aimed at improving this belief in self-ability can help improve the recovery trajectory of individuals after knee arthroplasty. ^6^

Our previous research in India demonstrated that, an individuals’ behaviour to adhere to the rehabilitation protocol was poor due to inability to perform the physical therapy without support, anticipated positive and negative belief about performing exercises, lack of motivation and socio-cultural factors such as family support, and difficulty in access to professional physiotherapists. ^7^ In line with Bandura’s theory of self-efficacy, we designed and developed a mobile-based health (mHealth) intervention using the behavior design thinking approach to support home-based rehabilitation. ^8^ The overarching aim of this intervention was to improve functional outcomes by improving self-efficacy to prescribed rehabilitation regimen. In Table 1, we demonstrate how each component of the TReAT intervention maps to the challenges identified in the formative phase. ^7^^,^^8^ The TReAT intervention consists of five interlinked components that provide information to patients about the recovery pathway following knee arthroplasty, enabling the patient to adhere to the prescribed exercises via reminders, motivational messages, and recorded exercise videos; a secure portal for one-to-one communication with health care providers via text, video, and audio calls; and self-monitoring through use of an e-diary. Furthermore, this intervention enables healthcare providers (surgeon and physiotherapist) to remotely monitor and prescribe personalized physical therapy plans. ^8, 9^ This intervention is delivered through the “TeleRehabilitation after knee ArThroplasty (TReAT) app” which consists of seven functionalities, exercise videos, e-diary, knowledge material, progress graphs, in-app messaging, in-app video consultation and a tool to self-measure knee range of motion. The details of the app functionalities to deliver the intervention are illustrated in **section 1 of the supplementary file.** ^10^

**Table 1.** Mapping the interventions with the barriers of adherence to rehabilitation (Adapted from Ref. 7).

Barriers/facilitators identified during formative phase of TReAT project	Capability Opportunity and Motivation-Behaviour (COM-B)	Intervention component & corresponding app feature
• Pain interference to perform the exercise • Lack of information on relationship between pain and exercise	Physical capability Reflective motivation	An e-booklet consisting of context specific information related to preparing oneself for surgery, knowing about recovery process, pain management, and important do and don’ts to safeguard the new knee **App feature:** e- **Education content**
• Inadequate skills to perform exercise • Difficulty to retain information on exercises & advice	Physical capability Psychological capability	Audio visual aids for patients to perform exercises for range of motion, muscle strength, balance, and function **App feature: Videos of exercise as prescribed by the physiotherapist**
• Lack of awareness about importance of exercises for recovery • Lack of motivation in patients for following the exercises	Psychological capability Reflective motivation Automatic motivation	Goal setting of desired activity level at the start of therapy. An e-diary to self-monitor pain, range of motion, knee function, activity level, and adherence to exercises. Motivational messages and remote video consultation with health care provider (surgeon and physiotherapist) **App feature: My Diary, Range of motion, Motivation message notifications depending on time since surgery and remote consultation**
• Poor accessibility or high cost to avail physiotherapy services and devices • Difficulty in commuting to the referral hospital	Environmental opportunity	Two-way synchronous and asynchronous messaging via audio, video, and text features with the health care provider (surgeon and physiotherapist) **App feature: Message & Appointment for remote consultation**
• Need for personalized continued support from health care provider after discharge to home	Environmental Opportunity	Facilitating continuum of care between health care provider (surgeon and physiotherapist) and patients by modifying treatment plan based on the progress. **App feature: Progress graphs, remote consultation and therapy planning**
• Need for physical and psychological support from family	Social opportunity Reflective Motivation	Family members/caregiver will be sensitized to recovery needs via education material, view progress and goals achieved by patient and be there during remote consultation **App feature: Family member interface**

The clinical and cost-effectiveness of this app-based rehabilitation support is being evaluated in an open-label, 1:1 randomized, superiority trial (TReAT trial) at two tertiary care hospitals. ^11^ Consenting individuals with the ability to use the basic functions of a smartphone and willing to visit at 3- and 6-months post knee arthroplasty were randomised to either the TReAT app plus usual care or usual care only. The intervention was provided for a minimum duration of 6 months. In India, usual care after knee arthroplasty consists of unsupervised home-based rehabilitation, which consists of provision of oral or written instructions at discharge and follow-up check-ups with the surgeon or physiotherapist as per individual need. Individuals may avail support from physiotherapy services at home or visit a clinic at their discretion. To understand the implementation fidelity, reach, any unanticipated additional activities or adaptations to the intervention, and the mechanistic pathway of various components of the TReAT intervention, we are conducting a process evaluation using a mixed-methods approach in accordance with the UK Medical Research Council process evaluation guidance for complex interventions. ^12^ In this study, we detail the process evaluation protocol for the TReAT trial.

### Objectives

• To evaluate implementation *fidelity* (how and extent of implementation with the original plan)
• To assess the *reach* (participants’ enrolment and retention) of the intervention, *dose* of the intervention *delivered and received*, and *usage* of the application
• To identify *contextual factors (barriers and facilitators)* for implementing this intervention
• To examine user *satisfaction and acceptability* of intervention components
• To examine the correlation between the extent of app function usage and expected behavioral changes *(mechanistic pathway)*

## Methods

### Conceptual framework

The process evaluation plan was developed using the six steps proposed by Saunders et al. ^13^ based on the theoretical framework by Linnan and Steckler. ^14^ The six steps include describing the intervention, defining what a complete and acceptable implementation would look like, developing a list of potential process evaluation questions, determining the methods for answering each of the questions along with the analysis & reporting. The process evaluation outcomes are based on the RE-AIM framework ^15^ and supplemented with the implementation outcome framework. ^16^ The additional outcomes are penetration and acceptability of the intervention.

A thematic illustration of the mechanism of change, using a logic model, is shown in Figure 1. We hypothesized that providing access to educational material from a credible source would improve patient knowledge about the recovery process and assist in returning to routine activities of daily living. System-generated notifications, self-monitoring of progress, and periodically communicating with healthcare providers (surgeon and physiotherapist) through app messaging and video consultation would improve their self-efficacy to adhere to the rehabilitation protocol. Personalized exercise therapy prescribed based on progress gauged either by remote consultation or by patient diary entry can help achieve better pain management and knee function. Thus, a portal for regular communication between healthcare providers and patients would aid in providing a continuum of care and help in motivating the patients to adhere to rehabilitation protocols during the recovery period.

**Figure 1.**
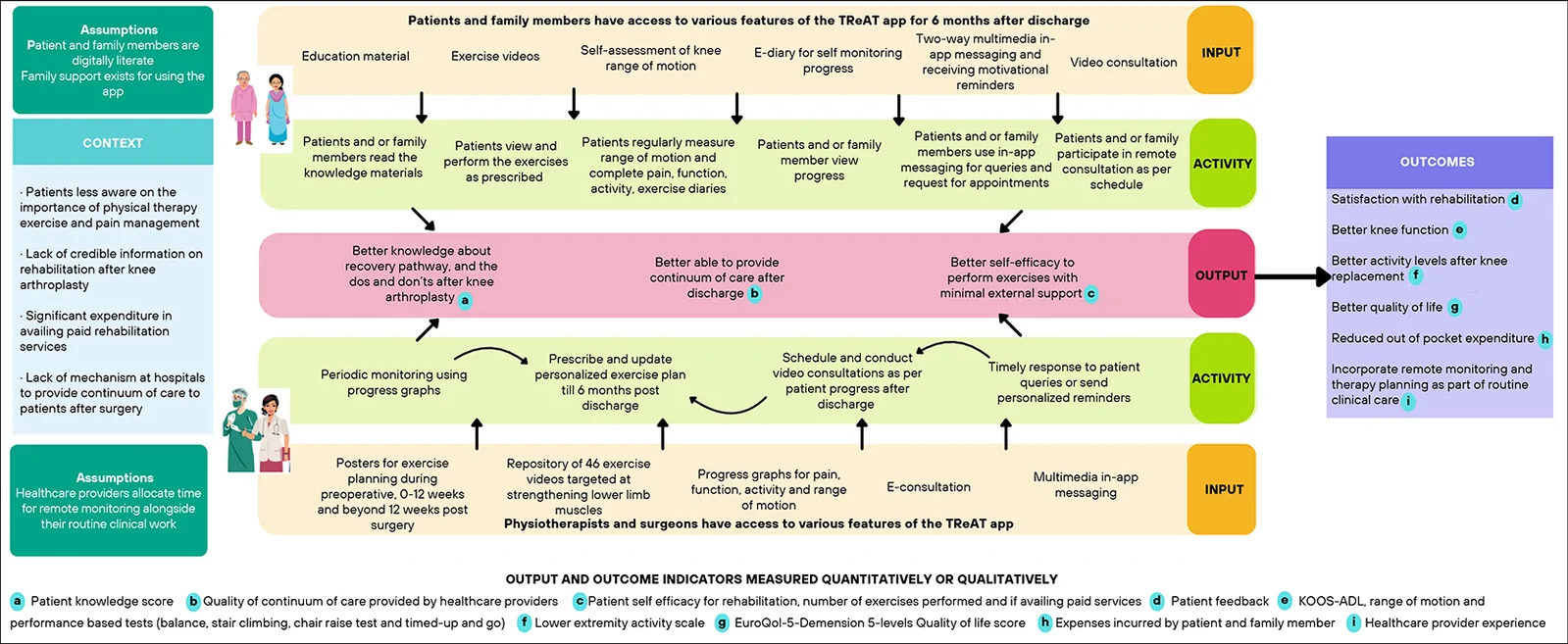
Intervention logic model. Legend- The figure illustrates the hypothesized mechanism of change enabled by the app-supported rehabilitation. The ‘Context’ is based on findings from literature review and formative phase of this project.

We defined ideal implementation of the intervention to include the following: (i) successful installation of the application by the end users with or without assistance from the research coordinator, followed by training on the use of different app components; (ii) healthcare providers use specific components of the app; for example, a physiotherapist and surgeon will monitor patient progress, update the therapy plan (minimum of 3 updates after discharge), and schedule video consultations (minimum 1 video consultation/per month in 0-3 months) as per patient requirements; (iii) patients and family members would be reading the educational material, referring to exercise videos, using the e-diary for self-monitoring (at least 50% as advised) and communicating with the healthcare providers (surgeon and physiotherapist) via messaging and/or video consultation would indicate that the intervention is acceptable.

### Study setting

The trial is being conducted at a public and private tertiary care hospital in Delhi, India, which will provide a contextual contrast in implementing the intervention. Individuals scheduled for or undergoing knee arthroplasty were eligible for the trial if they owned a smartphone and were able to use their basic functionalities. Furthermore, they should be willing to visit the hospital at 3 and 6 months after enrolment. The first participant was enrolled in June 2024 and recruitment ended in December 2025. The overall planned trial sample size was 300 participants.

### Study design

We will use a mixed-methods approach to collect data, analyze and interpret the results ^17^ using a convergent parallel design with nested sampling ( Figure 2). The rationale for using the mixed-method approach is treatment integrity and significance enhancement. ^17^ Under treatment integrity, the purpose is to identify discrepancies between planned and actual implementation of the intervention and identify barriers and facilitators in using the app. Under significance enhancement, the purpose is to understand the mechanism by which the intervention components lead to the outcome (triangulation) and to assess the adoption of the intervention by comparing results obtained from quantitative and qualitative methods (complementarity). The participants for the process evaluation were nested within those participating in the trial and sampling strategy is elaborated in the subsequent sections.

**Figure 2.**
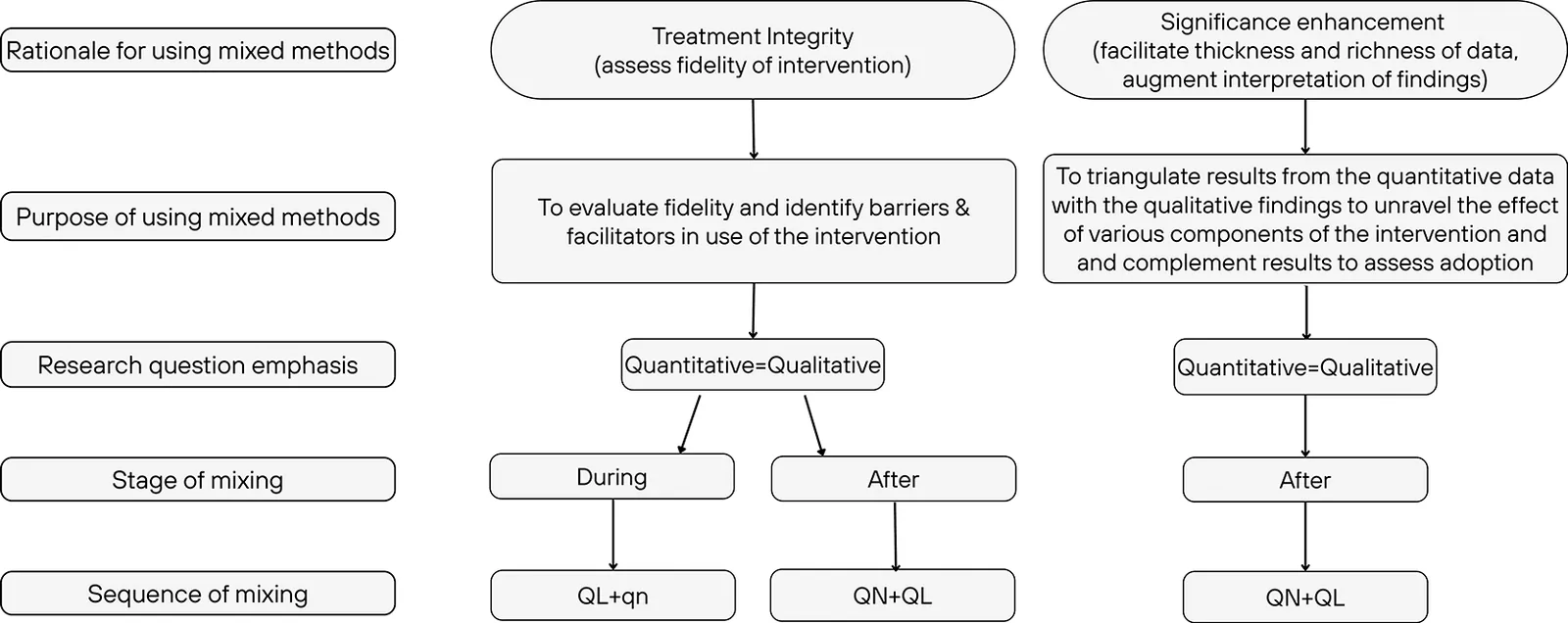
Rationale and purpose of mixed methods approach. Legend- Note: QN= quantitative, QL=qualitative, ‘+’= concurrent, ‘uppercase’= dominant, ‘lowercase’= less dominant. Reference: Collins KM, Onwuegbuzie AJ, Sutton IL. A model incorporating the rationale and purpose of conducting mixed-methods research in special education and beyond. Learning disabilities: A contemporary journal. 2006;4(1):67-100.

### Outcomes and data sources

We will assess reach, penetration, implementation fidelity, effectiveness, acceptability of the intervention, adoption and maintenance in the context in which the trial is conducted as process evaluation outcomes. Definitions of these outcomes are available **in section 2 of the supplementary file.** ^10^ We will measure the output level indicators such as better knowledge about recovery process, number and quality of contacts between patient and health care provider and better self-efficacy for doing the rehabilitation exercises. These indicators will help us understand the potential association between use of intervention components and outcomes. All randomized participants will contribute to reach and effectiveness; however, only the TReAT app group will contribute to implementation fidelity, and acceptability. Healthcare providers (surgeons and physiotherapists) will contribute to the understanding of TReAT intervention adoption during the trial and in routine practice. The source of data for the outcomes will be backend analytics of the mobile app, interviews with patients, physiotherapists, and surgeons, and researcher notes and observations ( Table 2).

**Table 2.** Description, measurement and analytic approach of the outcomes.

Domain of inquiry	Key questions (methodology)	What is measured and how is measured?	Analysis
Reach	What was the extent to which those who were approached were eligible? (Quantitative)	Number approached, ineligible, refused and recruited with their demographic characteristics (age, gender, literacy and location of residence) using a patient screening log	Reach=number recruited in the trial/total approached
Penetration	What was the extent to which those who were eligible were recruited? (Quantitative)	Number eligible, recruited and refused	Penetration=number recruited in the trial/total eligible
Effectiveness	What was the difference in knee function and quality of life between intervention and control group? (Quantitative)	Self-reported Knee function score (KOOS-ADL) and quality-of-life (EQ-5D-5L) of trial participants in both groups measured at 3 and 6 months	Group comparison in KOOS-ADL and quality of life scores at 3 and 6 months
Pathways to outcome	Which components of the intervention could have impacted the outcome? Does provision of education material and exercise videos improve knowledge about post-op care, rehabilitation adherence and number of exercises performed? Does knowledge and adherence vary by extent of app use? Is greater patient knowledge/rehabilitation adherence associated with better functional outcomes? (Quantitative and Qualitative)	Extent of app used from the fidelity tool (quantified researchers note and app analytics). Patients’ knowledge about recovery (14-item questionnaire), rehabilitation adherence (22-item questionnaire) and exercise performance using a predefined checklist (objectively measured). These are study specific tools administered to all participants at 3 months.	Group comparison of patient knowledge score, rehabilitation adherence score and proportion of exercise performed at 3 months. Difference in these scores by use of the app components. Correlation of these scores with KOOS-ADL scores by group.
Fidelity	Were intervention components delivered/used as planned? (Qualitative and Quantitative)	Intervention components used by end users captured from researcher notes, app analytics data compiled using a fidelity tool	Frequency of use of each component of app by end users.
Adoption	How did the healthcare providers uptake the intervention during the study? (Qualitative and Quantitative)	Healthcare provider engagement with the application for providing post knee arthroplasty rehabilitation care captured via unobtrusive observations, healthcare provider and patient interviews, app analytics	Proportion of patients for which app components were used in each context and compare it with the organisational and provider level barriers and facilitators identified. Quality of remote interactions using event analysis
Acceptability	How satisfied were the end users with the intervention? (Qualitative)	Semi-structured interviews of end users to know satisfaction with navigating the app, satisfaction with the contents of the intervention and its perceived relevance	Thematic analysis of perceived satisfaction, usefulness, relevance, and barriers to use among patients, family members and healthcare providers.
Context	Did any individual level characteristics and health sector (public and private) influence implementation at each site? What were the context specific adaptations that were made to address emerging challenges? (Qualitative)	Semi-structured interview of healthcare provider at the end of the study and researcher notes maintained throughout the study	Thematic analysis for contextual factors at organisational, provider and patient level to explain the implementation fidelity.
Maintenance	What is the healthcare providers’ perception on the potential for sustained and integration of the TReAT intervention into routine rehabilitation care beyond the trial? (Qualitative)	Semi structured interviews with healthcare providers towards the end of the trial to assess health care providers buy-in and opinions on the potential for use of the intervention in routine care	Thematic analysis for contextual factors at organisational and provider level.

Backend analytics from the mobile app: Participants’ and healthcare providers’ usage and adherence to the intervention will be assessed using data from the backend app analytics. The data include the number of times, the patients complete the diary (pain, exercise, knee function, and activity), the exercise prescription is modified by the healthcare provider, and video consultations scheduled. Use of the app by family members, number of times exercise videos are viewed, information about accessing the educational material, frequency of using the messaging feature, and viewing of the progress graphs will not be available in the app analytics. However, these will be captured using structured researcher notes described in the subsequent section.

Screening log: A screening log maintained by the research coordinator at each study site will document the number of patients scheduled for knee arthroplasty, number approached, enrolled, reasons for ineligibility, and refusal to participate, along with age, sex, literacy, and state of residence.

Researcher notes: The research coordinator will document information related to app installation, healthcare provider, patient and family member engagement with the app and the quality of video calls in a structured diary using an Excel workbook from direct observations and periodic checks in calls made to the trial participants ( **section 3 & 4 of the supplementary file** ^10^). To gauge the quality of healthcare provider’s interaction during video consultations unobtrusive observation will be made by the research coordinator and documented as notes.

End-user interviews will be conducted to explore participant experience with remote consultation, usefulness of knowledge material, satisfaction with the app, and use of the app to support rehabilitation. The interviews will be conducted with a random subsample of participants in the intervention group stratified by app usage. Participants will be classified as full, partial, or non-users based on the number of components of the app used. Participants who use more than five of the seven components of the app will be classified as full users. Partial users will be those who use at least one to five components, and non-users will be those who failed to install or did not use the app even after installation. Physiotherapists and surgeons participating in the trial will be interviewed at the end of the study to obtain feedback on their experience in app usage and the potential for uptake of this intervention in routine rehabilitation care. Semi-structured interview guides for the interviews are available in **section 5 & 6 of supplementary file.** ^10^

Study specific questionnaires: Patients’ knowledge about recovery, the type of exercises being performed, experience during rehabilitation period, and self-efficacy for adhering to the rehabilitation exercises will be measured at 3 months in both groups to understand the mechanisms through which the intervention may have an impact on the functional outcomes ( **section 7, 8 & 9 of supplementary file** ^10^). These questionnaires were developed specific for this intervention and have not been evaluated for their psychometric properties.

### Ethical considerations

The trial protocol, including the process evaluation activities, was approved by the ethics committees of the sponsor institute and clinical sites. Written informed consent was obtained from all study participants.

### Data collection

Quantitative component: Trained research coordinators will collect data on knowledge, exercise performance, and rehabilitation adherence at the 3-month follow up using study-specific questionnaires. Data will be recorded on paper forms and entered into REDCap, a web-based electronic data capture system with built-in validation checks and a query management system. Researcher notes and observations will be collated in a structured Excel spreadsheet ( **section 10 of supplementary file** ^10^ **)**.

Qualitative component: Interviews will be conducted during the 6-month follow-up from purposively selected participants in the app-based rehabilitation group (users, partial users, non-users based on the app analytics and researchers’ notes) using a semi-structured topic guide to explore the rehabilitation experience and barriers and facilitators to intervention use. Al healthcare providers involved in delivering the intervention (n=4) will be interviewed to explore their experience in using the app and their perceptions of the potential for this intervention in routine rehabilitation care. A researcher trained in qualitative interview methods, will conduct and audio-record interviews either face-to-face or telephonically. If participants are uncomfortable or refuse to be audio recorded, notes will be taken during the interview by the researcher. Participants for qualitative interviews will be selected using purposive, stratified sampling guided by the principle of information power. ^18^ The sample will include participants from the TReAT app group across predefined user categories: full users, partial users, and non-users, with attention to variation by study site and relevant sociodemographic characteristics where feasible. Information power will be used because the qualitative inquiry has a focused aim, is informed by a conceptual framework and semi-structured interview guides, and targets participants with direct experience of intervention implementation and use. Sampling will continue until sufficient information is obtained to address the process evaluation questions across user categories and site contexts, within the pragmatic constraints of the trial. All healthcare providers involved in delivering the intervention will be invited for interview because they represent a small, information-rich group central to understanding adoption and maintenance.

### Data management

Data from questionnaires related to process evaluation are entered into the trial database developed in REDCap and will follow the same procedures for ensuring data quality, confidentiality, and security as for the effectiveness outcomes, as described in detail in the trial protocol. ^11^ Data from backend app analytics will be periodically extractable by the trial team without personal identifiers. Qualitative interviews will be transcribed and translated by one member of the research team and cross-checked by another member of the team for correctness and appropriateness. All patient identifiers will be removed at the time of the transcription. Data related to the screening log and researcher notes maintained in an Excel sheet will be stored only for a definite duration after trial completion. The data on the app platform will be destroyed five years after the completion of the trial. The rest of the data will be stored securely with restricted access on the sponsor’s server, as per institutional policy. The paper forms and informed consent will be safely secured in a cabinet with restricted access at the sponsor institute as per the institutional policy.

### Data analysis

Reach & Penetration: Data from the screening log will be analysed descriptively using Stata (Stata Corp). The proportion of non-participants and participants recruited from the total approached (reach) and recruited of the total eligible (penetration) will be presented along with sociodemographic characteristics such as age, gender, education and place of residence.

Effectiveness: The primary effectiveness outcome is self-reported knee function (KOOS-ADL) and the secondary effectiveness outcomes are Lower Extremity Activity Level (LEAS), performance-based measures and quality of life (EQ-5D-5L). These outcomes will be compared at 3 and 6 months after randomisation between the TReAT app and the usual care group. Further details about effectiveness outcomes and analysis can be found in the published trial protocol ^11^ and the statistical analysis plan. ^10^

Adoption: To gauge healthcare provider engagement with the application we will report the proportion of patients for which app components were used in each context (private and public hospital) and triangulate this with the organisational and provider level barriers and facilitators identified. Further the quality of remote consultation will be summarized using event analysis.. The unit of analysis will be a successful video consultation. The items assessed will include ease of joining, audio and video quality, involvement of the caregiver, duration of consultation, resolution of any query enquired by the patient and family member, and assessment of-wound, range of motion, posture, and gait. Event frequencies and proportions will be summarized descriptively and interpreted in relation to intervention adoption.

Implementation fidelity: Extent of participant and family member engagement with prescribed TReAT app components (using the e-reading material, use of diary, use of messaging feature, video consultation, using videos to perform exercises, measuring knee range of motion using the app) from researcher notes and app backend analytics will be consolidated into one dataset and then presented using descriptive summaries. At interpretation, quotes from researcher notes and in-depth interviews will be utilized to demonstrate why certain components of the app were used or not used.

Maintenance: To assess the perceived potential for sustained use of the TReAT intervention beyond the trial period, semi-structured interviews with healthcare providers will be thematically analysed to explore organisational and provider level factors influencing its integration into routine rehabilitation care.

Pathways to outcome: We will explore the hypothesised pathways linking TReAT app components to functional outcomes by examining intermediate outputs at 3 months, including patient knowledge, rehabilitation adherence and types of exercise performed. These outputs will be compared descriptively between the TReAT app group and usual care group, and within the TReAT app group by level of use of relevant app components such as educational material and exercise videos. Associations between KOOS-ADL scores and intermediate outputs will be examined by group.

Qualitative data on rehabilitation experience will be thematically analysed to contextualise the quantitative findings and explain how the intervention components may have influenced outcomes. Transcripts will be analysed using an inductive-deductive thematic approach. The deductive component is justified by the predefined process evaluation outcomes and interview domains, while the inductive component will allow unanticipated barriers, facilitators, adaptations, and contextual influences to emerge from participants’ accounts. An initial coding framework will be developed by two independent researchers from the study objectives, interview guides, implementation outcomes, and familiarisation with a subset of transcripts. The researchers will then compare and discuss the codes, and a set of codes will be finalized through consensus within the research team. The transcripts will be coded using the agreed codes by one researcher. To minimize subjective coding bias and ensure analytic rigor, emerging codes, themes, and subthemes will be regularly reviewed and discussed with the wider research team, and any discrepancies or refinements will be resolved through team consensus. The final themes and their interpretation will be agreed upon by the research team. Themes will be compared across user categories and hospital settings. Quantitative and qualitative data will be analysed separately and triangulated during the interpretation. Findings will be compared across the key process evaluation domains as described in Table 2 to examine convergence, complementarity, and divergence. Any discordant findings will be examined in relation to participant experiences, contextual & implementation factors and reported transparently to inform interpretation of the results.

## Discussion

This paper describes the design of a mixed-method process evaluation protocol for a multicomponent behavioural intervention conducted alongside a randomized controlled trial. ^11^ The TReAT intervention was designed to support recovery and rehabilitation of patients undergoing knee arthroplasty. There is an increasing trend of mHealth interventions being developed and evaluated for patient management in clinical contexts, but details about intervention fidelity, adoption, and implementation ability are often understudied or under-reported, limiting the interpretation of trial findings. ^19, 20^

This evaluation, guided by the Linnar and Strickler framework ^14^ will help us understand the results of the study, irrespective of the strength and direction of our findings. We will also be able to answer questions regarding the generalizability and future scalability of this intervention in populations with less proficiency in using smartphones and low-resource settings. The remote monitoring approach using mobile applications may not be novel in the global context ^21^ however, the contextual factors influencing the adoption of the remote monitoring approach among patients and healthcare providers will add valuable information for future work in this space in a low-middle-income country context.

### Strengths and limitations

The strengths of this evaluation lie in its comprehensive planning and clear reporting of the mixed-methods approach. We will be reporting the results of this evaluation following the standard reporting guidelines of mixed-methods studies for rehabilitation and health sciences. ^22^ The integration of qualitative and quantitative methods will provide a more nuanced understanding of the findings and lead to better use of the data collected. A limitation of the study is that the findings will be more applicable to tertiary care referral hospitals in the North Indian context. Second, reliance on researcher notes and self-reported data may introduce reporting bias. Finally, we are only proposing correlation between outcome and outputs and not a mediation analysis to explore the mechanistic pathways to outcome owing to small sample size.

## Conclusion

This protocol is an attempt to improve the planning, analysis, and interpretation of process evaluation, alongside a clinical trial evaluating an mHealth intervention for remote monitoring. The results of this trial will inform future mHealth interventions in physical rehabilitation spaces in the context of the elderly population and lower-middle-income countries.

## Patient and public involvement

There was no patient or public involvement in the design or conduct stages of the process evaluation of this trial.

## Data Availability

No data are associated with this article The supplementary file and completed SPIRIT 2025 checklist can be found in **Open Science Framework** in a folder named “Process evaluation protocol” in the project ‘Telerehabilitation for post knee replacement monitoring (TReAT project).’ This file can be accessed at https://doi.org/10.17605/OSF.IO/AQGRM ^10^ Description of data: This supplementary file contains a description of the intervention, data collection tools, and definitions of the implementation outcomes.
1. eFigure: TReAT app to deliver the intervention 2. eTable: Definitions of process evaluation outcomes 3. Structured format to capture researcher’s notes for the intervention group 4. Structured format to capture researchers note-usual care group 5. Semi-structured interview of patients or family member-intervention group 6. Semi-structured interview guide for health care providers (surgeons and physiotherapists) 7. Questionnaire to assess patients’ knowledge about recovery 8. Questionnaire to assess rehabilitation adherence 9. Questionnaire to assess exercise performance 10. Fidelity tool Data are available under the terms of the Creative Commons Zero “No rights reserved’ data waiver (CC0 1.0 Public domain dedication).
