# Supplementary material for: Process evaluation protocol of a trial evaluating app-based post knee arthroplasty telerehabilitation strategy in India
Source: Wellcome Open Res. 2026 Sep 29;11:207. Originally published 2026 Apr 8. [Version 3] doi: 10.12688/wellcomeopenres.26007.3 (PMC13560626; doi:10.12688/wellcomeopenres.26007.3)
Supplement: Peer review history

# Reviewer report: Reviewer response for version 3

**DOI:** 10.21956/wellcomeopenres.30697.r171126

**Published:** 2026-10-05

**Authors:** Nirali Shah

**Affiliations:** 1 University of Michigan, Ann Arbor, USA

Thank you for the opportunity to review. The authors have addressed all my comments appropriately.

Is the study design appropriate for the research question?

Yes

Is the rationale for, and objectives of, the study clearly described?

No

Are sufficient details of the methods provided to allow replication by others?

No

Are the datasets clearly presented in a useable and accessible format?

Yes

Reviewer Expertise:

Digital health rehabilitation

I confirm that I have read this submission and believe that I have an appropriate level of expertise to confirm that it is of an acceptable scientific standard.

# Reviewer report: Reviewer response for version 2

**DOI:** 10.21956/wellcomeopenres.30291.r164722

**Published:** 2026-08-28

**Authors:** Nirali Shah

**Affiliations:** 1 University of Michigan, Ann Arbor, USA

Dear authors,

 Thank you for the substantial revisions. It is clear that a lot of thought and effort has gone into putting this together. The proposal is scientifically rigorous but needs some clarifications and minor revisions. Please see details below:

 Abstract

- Please tone down language related to correlations determining “mechanistic pathways.” Correlations can demonstrate associations and generate hypotheses regarding possible mechanisms, but they cannot establish causal mechanisms. Describe these analyses as exploratory associations/pathway hypotheses unless a causal mediation approach is planned.

Introduction

- In Table 1, if pain is the primary barrier being addressed by the educational component, consider describing the pain-related educational content first, followed by the broader perioperative/recovery information, so that the proposed intervention-target relationship is clearer.
- Where the intervention refers to communication or remote consultation with a “health care provider,” specify the relevant clinician(s) (e.g., physiotherapist, surgeon).

Methods

- Please specify how key outcomes are measured, i.e, are they self-reported, objectively measured, researcher-observed, or obtained from backend analytics?
- Please justify the thresholds defining full users (>5 components), partial users (1–5), and non-users. The categories are arbitrary and are important because they are used for qualitative sampling and subgroup comparisons.
- Backend analytics still do not capture several intervention components, including educational material access, exercise-video viewing, messaging, progress-graph viewing, and family-member use. Please explain how reliance on researcher notes/observations for these components may affect completeness and validity of fidelity and usage estimates.
- Family members/caregivers are included in the intervention and fidelity/acceptability descriptions. Please make their role in the evaluation explicit and consistent, including how their engagement and experiences will be sampled and analyzed.
- The manuscript now states that qualitative and quantitative findings will be integrated during interpretation to assess convergence, complementarity, and divergence. Please specify the formal mixed-methods integration framework or procedure that will guide this integration (for example, how datasets will be brought together, whether joint displays will be used, and how discordant findings will be handled). Simply stating that findings will be compared or triangulated is not sufficient
- The qualitative analysis description is not consistent. First, it states that transcripts will be coded independently by two researchers, but then states that a trained researcher will manually code initial transcripts. Please clarify exactly how many coders will code each transcript and at what stages. Also, one coder alone is not sufficient to adequately address subjective coding bias

- The proposed “event analysis” of remote consultations requires a clearer methodological description or citation. Please define how events are coded, what the unit of analysis is, how observer ratings are standardized, and how resulting quantitative summaries will be interpreted.

Minor Changes

- The manuscript is a protocol but shifts between future and past tense. Please use tense consistently and reserve past tense for activities that were genuinely completed before publication
- Please use “behavior/behaviour” consistently according to the journal’s preferred style

- Figures 1 and 2 are extremely pixelated and unreadable in the tracked manuscript. Please replace it with a high-resolution figure
- Remove unnecessary italics and duplicated bullet symbols in the Objectives section and perform a careful formatting clean-up throughout.
- Quotation marks around methodological terms such as “treatment integrity,” “significance enhancement,” “triangulation,” and “complementarity” are distracting unless they indicate terminology quoted directly from a source. Please remove them or cite their use.
- Please cite the appropriate reference for “information power approach”

Is the study design appropriate for the research question?

Yes

Is the rationale for, and objectives of, the study clearly described?

No

Are sufficient details of the methods provided to allow replication by others?

No

Are the datasets clearly presented in a useable and accessible format?

Yes

Reviewer Expertise:

Digital health rehabilitation

I confirm that I have read this submission and believe that I have an appropriate level of expertise to confirm that it is of an acceptable scientific standard.

# Reviewer report: Reviewer response for version 1

**DOI:** 10.21956/wellcomeopenres.28625.r158042

**Published:** 2026-07-02

**Authors:** Nirali Shah

**Affiliations:** 1 University of Michigan, Ann Arbor, USA

Dear authors,

 The manuscript addresses an important implementation question and is generally well organized. However, several conceptual and methodological issues should be addressed to improve the clarity and rigor of the proposed protocol. In particular, the primary aim of the study becomes unclear as additional objectives and outcomes are introduced throughout the manuscript. Furthermore, the theoretical framework, implementation frameworks, proposed outcomes, and analytical approach are not always well aligned. A clearer conceptual framework and stronger alignment between the study objectives, methods, and planned analyses would substantially strengthen the protocol. Please find specific feedback below:

 Abstract

- The abstract states that descriptive summaries will be presented for effectiveness outcomes. However, effectiveness outcomes generally require inferential statistical analyses rather than descriptive summaries alone. Please clarify whether inferential analyses are described elsewhere (e.g., in the primary trial protocol) or summarize the planned analytical approach.
- The implementation outcomes (reach, fidelity, adoption, maintenance, etc.) are introduced without explanation. Consider briefly defining these metrics to improve accessibility for readers unfamiliar with implementation science terminology.
- The abstract introduces "mixing" but does not describe how qualitative and quantitative data will actually be integrated. Consider briefly describing the integration strategy rather than simply stating that mixing will occur.

Introduction

- The rationale begins by emphasizing rehabilitation adherence but later introduces additional objectives such as detecting slow recovery (Figure 1) and reducing clinical visits without explaining how these relate to the original theoretical framework. Consider strengthening the conceptual flow of the introduction.
- The intervention is described as being based on Bandura's self-efficacy theory; however, the manuscript does not clearly explain how each intervention component operationalizes this theory. For example, it is unclear how reminders, educational content, remote monitoring, provider messaging, and personalized exercise prescription are expected to improve self-efficacy. Explicitly mapping intervention components to theoretical constructs would strengthen the rationale. Also, the introduction appears to introduce new intervention objectives throughout the narrative (e.g., adherence, slow recovery detection, reducing clinical visits, improving knowledge, improving self-efficacy). Please be specific about hypothesized mechanism and consolidating these objectives into a single coherent conceptual framework.
- The introduction refers to the e-diary as a self-management component, although it appears to function primarily as a self-monitoring tool. Please clarify this conceptual distinction or justify why the e-diary represents self-management rather than self-monitoring. Also, later on in the paper e-diary is noted as a self-monitoring tool. Please be consistent throughout.

Methods

- Conceptual Framework – The authors have referenced multiple frameworks (Linnan & Steckler, RE-AIM, Proctor implementation outcomes, and MRC guidance), but the rationale for combining these frameworks is not clearly described. Please clarify the specific role each framework plays within the process evaluation.
- The logic model introduces several constructs (e.g., awareness, knowledge, goal setting) that were not clearly developed in the introduction. Consider aligning the conceptual framework more closely with the theoretical rationale presented earlier.
- The mixed-methods approach requires greater methodological detail. While the manuscript states that data will be integrated during collection and interpretation, it does not explain how qualitative and quantitative findings will be integrated, how conflicting findings will be reconciled, or whether joint displays or other recognized mixed-methods integration strategies will be used.
- The rationale for using a convergent parallel mixed-methods design is appropriate; however, the manuscript should provide greater detail regarding the mixed-methods integration strategy. Specifically, how will qualitative findings explain quantitative findings? How will conflicting findings be reconciled? Will joint displays or other formal integration methods be used?
- The rationale for nested sampling should be explained more clearly, particularly why qualitative interviews are limited to intervention participants.
- Several implementation constructs (e.g., fidelity, usage, adoption, acceptability, appropriateness, maintenance) appear conceptually overlapping. Please provide clearer operational distinctions between these constructs and explain how each will be analyzed separately.
- The rationale for classifying participants as full users (>5 components), partial users (1–5 components), and non-users appears arbitrary. Please justify these thresholds.
- Several important intervention components (e.g., educational material viewed, messaging frequency, progress graph use, family member engagement) are not captured through backend analytics. Please discuss how these missing data may affect assessment of intervention fidelity and usage.
- Family members are included within the intervention logic model (supplementary material) and are described as end users, yet their experiences are minimally incorporated into the evaluation plan. Please clarify whether family member engagement will be evaluated.
- The manuscript refers to study-specific questionnaires for knowledge, adherence, and exercise performance. Please provide evidence regarding the development, content validity, and reliability of these instruments or direct readers to where this information can be found.
- The statistical analysis plan appears incomplete. Although effectiveness outcomes, mechanistic pathways, and between-group comparisons are described throughout the manuscript, the Data Analysis section primarily presents descriptive summaries and app usage statistics. Please clarify the planned inferential analyses for effectiveness outcomes (e.g., estimation of treatment effects, confidence intervals, regression models, handling of repeated measures, and missing data), or explicitly state that these analyses are described in the primary trial protocol.
- The manuscript repeatedly refers to mechanistic pathways but does not clearly define the proposed mechanisms or causal assumptions underpinning the intervention. The proposed analyses consist largely of descriptive summaries and simple correlations, which are insufficient to evaluate mechanisms. Please clarify the hypothesized causal pathways, specify the proposed mediators (e.g., knowledge, self-efficacy, adherence), and describe how these pathways will be evaluated. Also, please clarify whether these analyses are intended to be exploratory and discuss whether more appropriate approaches (e.g., mediation analyses) are planned.
- Please add additional methodological detail in the qualitative analysis section, including development of the coding framework, coder training, procedures for resolving disagreements, reflexivity, maintenance of an audit trail, and the rationale for using an inductive-deductive approach.
- Please clarify whether interviews will continue until data saturation or whether an information power approach will be used and provide justification for the chosen sampling strategy.

Is the study design appropriate for the research question?

Yes

Is the rationale for, and objectives of, the study clearly described?

No

Are sufficient details of the methods provided to allow replication by others?

No

Are the datasets clearly presented in a useable and accessible format?

Yes

Reviewer Expertise:

Digital Health and Rehabilitation

I confirm that I have read this submission and believe that I have an appropriate level of expertise to confirm that it is of an acceptable scientific standard, however I have significant reservations, as outlined above.

# Response

**Published:** 2026-09-22

**Authors:** Niveditha Devasenapathy

**Affiliations:** Academic Trial Clinical trial Unit, The George Institute for Global Health India, New Delhi, Delhi, India

**Dear reviewer,** Thank you for the constructive comments. Please find our point-to-point responses. Thank you.

**Abstract**

1. Please tone down language related to correlations determining “mechanistic pathways.” Correlations can demonstrate associations and generate hypotheses regarding possible mechanisms, but they cannot establish causal mechanisms. Describe these analyses as exploratory associations/pathway hypotheses unless a causal mediation approach is planned.

***Response*** *: We have updated the text.* *Old text-To explore mechanistic pathways correlation between functional outcomes, knowledge score and rehabilitation self-efficacy will be examined.* *Updated text- To explore potential association between use of intervention components and outcomes, correlations between functional outcomes, knowledge score and rehabilitation self-efficacy will be examined.*

**Introduction**

1. In Table 1, if pain is the primary barrier being addressed by the educational component, consider describing the pain-related educational content first, followed by the broader perioperative/recovery information, so that the proposed intervention-target relationship is clearer.

***Response:*** *The order in which the educational content is described in Table 1 reflects the sequence in which topics are presented in the e-booklet. We retained this order to reflect how the educational material was presented to participants, while ensuring that pain management is identified as a key component of the intervention.*

1. Where the intervention refers to communication or remote consultation with a “health care provider,” specify the relevant clinician(s) (e.g., physiotherapist, surgeon).

***Response:*** *We have updated the text to specify the relevant healthcare provider ‘surgeon and physiotherapist’ involved in the intervention.*

**Methods**

1. Please specify how key outcomes are measured, i.e., are they self-reported, objectively measured, researcher-observed, or obtained from backend analytics?

***Response:*** *The details of how each outcome is measured is provided in column 3 of Table 2 and in the ‘outcomes and data sources’ section of the manuscript. *

1. Please justify the thresholds defining full users (>5 components), partial users (1–5), and non-users. The categories are arbitrary and are important because they are used for qualitative sampling and subgroup comparisons.

***Response:*** *The user categories were defined to reflect intervention core functions. Of the total 7 components, (messages, video consultation, e-diary, progress graph, exercise videos and range of motion) the diary and progress graphs are interrelated, and messaging & video consultation are mode of communication where messaging is optional and conditional. Therefore, we considered use of more than five components should be considered as user, use of one to five components as partial user.*

1. Backend analytics still do not capture several intervention components, including educational material access, exercise-video viewing, messaging, progress-graph viewing, and family-member use. Please explain how reliance on researcher notes/observations for these components may affect completeness and validity of fidelity and usage estimates.

***Response:*** *We agree some of the components were not measurable using backend analytics. While this might seem like a limitation, measures were taken capture these in an unobtrusive manner. The researcher notes were recorded using a structured template and completed after every interaction with the trial participant and family member. The information that may not have been captured during one interaction could often be documented in subsequent contacts. Further, there is no other way we could have collected the participant’s experience. To ensure data quality, notes were regularly reviewed by the trial manager for completeness. In addition, an iterative feedback process during the early stage of data collection, helped to refine the documentation and improve the richness and quality of the notes over time.*

1. Family members/caregivers are included in the intervention and fidelity/acceptability descriptions. Please make their role in the evaluation explicit and consistent, including how their engagement and experiences will be sampled and analyzed.

***Response:** We do have sections in the researchers notes (already mentioned in the manuscript) to identify if the family member read the education material, assisted in video consultations and messaging the health care provider. Further, the qualitative data about rehabilitation experience will also inform the level of involvement of the family member in the recovery phase.  *

1. The manuscript now states that qualitative and quantitative findings will be integrated during interpretation to assess convergence, complementarity, and divergence. Please specify the formal mixed-methods integration framework or procedure that will guide this integration (for example, how datasets will be brought together, whether joint displays will be used, and how discordant findings will be handled). Simply stating that findings will be compared or triangulated is not sufficient.

***Response*** *: Thank you for this suggestion. We have clarified the mixed-methods integration approach in the data analysis section. Quantitative and qualitative data will be analysed separately and triangulated during the interpretation. Findings will be compared across the key process evaluation domains to examine convergence, complementarity, and divergence. Any discordant findings will be examined in relation to participant experiences, contextual & implementation factors and reported transparently to inform interpretation of the results.*

1. The qualitative analysis description is not consistent. First, it states that transcripts will be coded independently by two researchers but then states that a trained researcher will manually code initial transcripts. Please clarify exactly how many coders will code each transcript and at what stages. Also, one coder alone is not sufficient to adequately address subjective coding bias

***Response:*** *Thank you for this comment. We have revised the description of the qualitative analysis in the data analysis section to improve clarity. An initial coding framework will be developed by two independent researchers from the study objectives, interview guides, implementation outcomes, and familiarisation with a subset of transcripts. The researchers will then compare and discuss the codes, and a set of codes will be finalized through consensus within the research team. The transcripts will be coded using the agreed codes by one researcher. To minimize subjective coding bias and ensure analytic rigor, emerging codes, themes, and subthemes will be regularly reviewed and discussed with the wider research team, and any discrepancies or refinements will be resolved through team consensus. The final themes and their interpretation will be agreed upon by the research team.*

1. The proposed “event analysis” of remote consultations requires a clearer methodological description or citation. Please define how events are coded, what the unit of analysis is, how observer ratings are standardized, and how resulting quantitative summaries will be interpreted.

***Response:*** *The unit of analysis will be a successful video consultation. The events assessed will include ease of joining, audio and video quality, consultation duration, assessment of-wound, range of motion, posture and gait, encouragement to use app functions by health care providers and caregiver involvement. Event frequencies and proportions will be summarized descriptively and interpreted in relation to intervention adoption. This has been updated in the data analysis section.*

 Minor Changes

1. The manuscript is a protocol but shifts between future and past tense. Please use tense consistently and reserve past tense for activities that were genuinely completed before publication.

***Response:*** *We have reviewed the manuscript for tense consistency and believe that the usage of tense in the manuscript is appropriate. Past tense is used for activities that had already been completed before publication, while future tense is used for activities that will be conducted during the study.*

1. Please use “behavior/behaviour” consistently according to the journal’s preferred style

***Response*** *: We updated and used ‘behaviour’ consistently throughout the manuscript.*

1. Figures 1 and 2 are extremely pixelated and unreadable in the tracked manuscript. Please replace it with a high-resolution figure

***Response***: *Figures 1 and 2 are provided in high resolution in the manuscript files submitted to the journal.*

1. Remove unnecessary italics and duplicated bullet symbols in the Objectives section and perform a careful formatting clean-up throughout.

***Response*** *: Thank you we have done the checks as suggested.*

1. Quotation marks around methodological terms such as “treatment integrity,” “significance enhancement,” “triangulation,” and “complementarity” are distracting unless they indicate terminology quoted directly from a source. Please remove them or cite their use.

***Response:*** *we have removed the quotation marks around the methodological terms.*

1. Please cite the appropriate reference for “information power approach”

***Response:*** *We have added the citation now to the manuscript.* *Roberts SJ, Saylor MA, Ivynian S, DiGiacomo M. Data saturation and information power: sample size in qualitative research-when is enough and how to assess? *Eur J Cardiovasc Nurs *. 2026;25(5):1069-1074. doi:10.1093/eurjcn/zvag046*

# Reviewer report: Reviewer response for version 1

**DOI:** 10.21956/wellcomeopenres.28625.r153021

**Published:** 2026-05-06

**Authors:** Pratik Phansopkar

**Affiliations:** 1 Musculoskeletal Physiotherapy, Bharati Vidyapeeth (Deemed to be University) School of Physiotherapy, Sangli, Maharashtra, India

The process evaluation protocol helps us understand how well an app-based telerehabilitation program works after knee replacement surgery in India. It looks at how patients use the app, stay engaged, and follow exercises. By identifying challenges and what works best, it ensures the program is effective, practical, and can be successfully expanded into everyday healthcare.

Is the study design appropriate for the research question?

Yes

Is the rationale for, and objectives of, the study clearly described?

Yes

Are sufficient details of the methods provided to allow replication by others?

Yes

Are the datasets clearly presented in a useable and accessible format?

Yes

Reviewer Expertise:

Musculoskeletal Physiotherapy, Post TKR & THR rehab.

I confirm that I have read this submission and believe that I have an appropriate level of expertise to confirm that it is of an acceptable scientific standard.
